# The impact of college experience on female students’ self-perceived employability in STEM majors

**DOI:** 10.3389/fpsyg.2023.1282934

**Published:** 2023-12-21

**Authors:** Wen Wen, Lu Zhou, Die Hu, Mingyu Zhang, Zehua Yan, Xiaofeng Tang

**Affiliations:** ^1^Institute of Education, Tsinghua University, Beijing, China; ^2^Hi-tech College, Xi’an University of Technology, Xi’an, China; ^3^Faculty of Education, Beijing Normal University, Beijing, China; ^4^Faculty of Humanities and Social Sciences, Beijing University of Technology, Beijing, China

**Keywords:** STEM education, female college students, Chinese higher education, hierarchical higher education system, gender disparities

## Abstract

**Introduction:**

The under representation of women in STEM fields is a persistent issue worldwide. In China, although women have made significant progress in pursuing STEM degrees in recent years, they continue to face challenges in the workforce. Given the importance of the self-perceived employability (SPE) of female STEM students in China, the research questions are: How do curriculum experience, extracurricular experience, and faculty supportive activities affect the SPE of female STEM students in Chinese universities? To what extent does university stratification affect the relationship between college experience and female STEM students’ SPE?

**Methods:**

We analyzed the 2018 data of the Chinese College Student Survey (CCSS) consisting of a sample of 59,066 students, and six focus group interviews.

**Results:**

The findings suggest that curriculum experience, extracurricular experience and faculty supportive activities have a positive impact on the SPE of female students, but the gender gap in SPE is still valid as reflected by the fact that women have lower SPE than men in each tier of universities and that men benefit more in terms of the increase in SPE from most types of college activities and support except academic ones.

**Discussion:**

This study reveals that the different tiers of universities in China affect female students’ SPE in different ways, and provides valuable evidence for academic as well as university administrators and policymakers regarding how college experience affect women and how university stratification can affect female students’ college experience and their career expectations and paths.

## Introduction

The gender gap between academic fields in higher education is still valid despite the worldwide increase in women’s access to higher education. Unequal gender representation is particularly obvious in STEM-related fields (STEM, refers to science, technology, engineering and mathematics); globally, only 30% of the female student population chooses STEM-related majors. Female students’ enrollment is particularly low in ICT (3%), the natural sciences, mathematics and statistics (5%), and engineering, manufacturing and construction (8%) (UNESCO, 2017). How women perceive themselves and evaluate their achievements in traditionally male-dominated fields such as STEM largely affects their choices in university and in the workforce (Broyles, 2009; Beede et al., 2011). Women often have reported lower perceived achievements than men even if they obtain higher demonstrated achievements (Astin and Sax, 1996). In many studies that have addressed the disadvantaged positions of women in STEM fields, women’s lack of self-confidence and lower evaluation of themselves are often regarded as the most important reasons that deter women from choosing or remaining in STEM majors and from seeking better employment when they enter the workforce (Hall and Sandler, 1982; Brainard and Carlin, 1998; Rothwell and Arnold, 2007; Sax, 2008; Qenani et al., 2014).

Self-perceived employability (SPE) refers to an individual’s perception of his or her likelihood of obtaining and maintaining sustainable employment appropriate to the individual’s qualification level (Ridgeway and Correll, 2004; Rothwell and Arnold, 2007; Vanhercke et al., 2014). SPE is closely related to individuals’ capability within the labor market to realize their potential through sustainable employment and may be a key goal for individuals in managing their careers (Rothwell and Arnold, 2007). However, the literature has some limitations. First, research on STEM women’s employability often considers the ways external factors, such as social bias, gender discrimination, and institutional type and ranking, shape their employability in the workforce (Ceci et al., 2009; Cho et al., 2009). However, this research rarely considers that college experience might be an important factor that shapes SPE in addition to other social influences and personal factors. Second, the impact of academic achievement and academic experience in college on female students’ SPE is unclear (Dovidio et al., 2012; Jackson and Wilton, 2017). Many universities provide opportunities for female students to develop their confidence in seeking employment (Wakefield et al., 2009), but there is a lack of knowledge of female students’ perceptions of the effectiveness of college activities in enhancing their SPE. Finally, most existing studies on SPE only focus on one or several universities (Qenani et al., 2014; Jackson and Wilton, 2017; Ma and Bennett, 2021), and there is little discussion of the levels of different universities in the national higher education system. In addition, due to limited accessibility of the data, the samples used in many published studies are too small to be nationally representative, and extensive sample studies covering different types of schools and various disciplines are lacking (Donald et al., 2018; Vargas et al., 2018; Wong et al., 2018; Monteiro et al., 2020). This research aims to fill the gap in the literature by examining the role of college experience in influencing women’s self-perceived employability.

The present study investigates the extent to which the college experience of female students in STEM majors influences their SPE in Chinese universities. It is critical to incorporate institutional contexts into the study of female students’ SPE. In relevant studies, it is quite common for students from higher-ranking universities to show higher perceived employability (Walters, 2004; Chen, 2016; Lee and Song, 2018) because university reputation contributes to the accumulation of human capital. However, in many cases, female students from elite universities have lower SPE than their demonstrated employability, as documented in a study on Australia’s Go8 (Group of Eight) elite universities (Jackson and Wilton, 2017) and a study on female graduates’ employability in the top 3 universities in Korea (Lee and Song, 2018). Students from elite universities might have higher expectations for employment, which affects their self-evaluation of their employability. It is yet to be determined whether upper-tier universities empower female students to develop self-confidence in their employability or whether these universities widen the gender gap in SPE.

In the case of China, its higher education system is stratified by several governmental projects to build world-class universities. ‘Project 985’ (research-extensive universities, called Tier-1 universities in our study) and ‘Project 211’ (research universities, called Tier-2 universities in our study) stratify the universities in larger society and send important signals to the job market. A degree from these universities can improve students’ employability, and applicants are considered in a hierarchical order with ‘Project 985′ graduates on top, followed by ‘Project 211′ graduates and then undergraduates from other universities (teaching-extensive and vocational-oriented universities, called Tier-3 universities in our study). There are significant differences among the three tiers of universities in terms of the resources and funding they can obtain from the government. According to 2013 statistics from the Ministry of Education, 39 Tier-1 universities received approximately 52% of government funding, 73 Tier-2 universities received 19%, and 1,100 Tier-3 universities received approximately 28% (Zeng and Li, 2014). Whether the hierarchy of Chinese universities differentiates the college experience for female students in STEM majors will be examined in this research. Additionally, the gender culture of China and Chinese universities is important to consider and helps in assessing the generalizability of the results of this study on the influence of college experience on female students’ self-perceived employability.

This research aims to address the issue that women and men may not be affected in the same way by their undergraduate experience, which includes experience in the curriculum, extracurricular activities, and faculty support related to career development. This study provides evidence of how female students’ perceptions of their employability in STEM careers are affected by their undergraduate experience. Moreover, this study sheds light on how the type of university can affect students’ employability in a hierarchical higher education system. This research can serve as a resource for academic affairs and student affairs professionals and other campus practitioners and for policymakers who are concerned with ways in which the return of college education might vary for students of different genders at different universities.

## A literature review on the self-perceived employability of female students in STEM majors

Research have shown that females were faced with more challenges in the job market that affect their employability in job market. Relatedly, due to unfavorable factors that prevent them from obtaining employment, female students often show lower self-perceived employability than males (Rothwell and Arnold, 2007). Self-perceived employability refers to the individual’s perception of his or her possibilities of obtaining and maintaining employment (Ridgeway and Correll, 2004; Rothwell and Arnold, 2007; Vanhercke et al., 2014). Males often have higher self-perceived employability before entering the market as they were aware of their gender advantage in the job market (Qenani et al., 2014).

Studies conducted in the context of China have recognized that the low self-perceived employability of female students in STEM fields was valid. They found that female students’ low self-perceived employability can also be associated with a few internal factors such as personalities and parents’ high expectations (Kong, 2007; Huang, 2011; Zhao, 2011; Li and Yang, 2016).

Furthermore, some scholars brought the influence of the college experience into the discussion of female students’ self-perceived employability. Academic achievements have been identified as a factor influencing self-perceived employability of female students in college; however, the impact is unclear. For example, Zhang and Zhen (2011) found that females students’ demonstrated advantages in academic performance during college did not instill confidence in them when it came to seeking employment. Moreover, student–faculty interaction was recognized as another important indicator of female’s self-perceived employability, as it may bolster or dampen women’s ambitions and self-confidence in areas that are traditionally viewed as masculine, such as science and technology (Hall and Sandler, 1982; Sax, 2008). Factors that may discourage women from persisting in STEM majors include the ‘chilly climate’ prevalent in the culture and pedagogy of STEM classes (Hall and Sandler, 1982) and dissatisfying experiences with the curriculum and interactions with faculty members and peers (Shapiro and Sax, 2011).

The positive relationship between undergraduates’ human capital and employability has been confirmed by many studies (Ben-Porath, 1967; Griliches, 1997; Smith, 2010). Students’ participation in work-integrated learning in college, such as internships or part-time work, can be important investments for the accumulation of human capital, which contributes to their employability in the job market (Becker, 1962) and enhances their confidence and perception of their own capabilities when preparing to enter the workforce (Jackson and Wilton, 2017). Ben-Porath (1967) formalized the investment-in-education process and noted that individuals add to their own human capital with their own time and other market resources. As such, university reputation largely develops the foundation for students’ human capital accumulation, and a diploma from a national flagship university serves an important ‘signaling’ or ‘screening’ role when graduates enter the workforce. Thus, students’ career management activities in college and their SPE should be closely related to the prestige of the universities they attend. How the reputation related factors of university can affect female STEM students’ SPE needs further investigation.

A few studies have documented that institutional characteristics play a crucial role in students’ sustained engagement in STEM majors (Wu and Li, 2020). Griffith (2010) found that students at institutions with more undergraduates than graduate students had higher rates of persistence in STEM careers because undergraduate-oriented institutions may create a more welcoming environment for students. An increase in the ratio of research to educational expenditures had a positive impact on persistence rates for men but a negative impact on persistence rates for women (Ehrenberg, 2010; Griffith, 2010). It is recognized in past studies that students’ college experiences shaped by institutional characteristics have different impacts on male and female students.

The existing body of research extensively explores the relationship between college experiences and self-perceived employability (SPE) among female students in science, technology, engineering, and mathematics (STEM) disciplines across various countries. However, a notable gap exists when applying these findings to the unique context of Chinese higher education that is characterized by a hierarchical structure, with universities classified into different tiers based on academic excellence and prestige. A study that captures the effects of different tiers of institutions on female’s SPE is needed in the Chinese higher education context. Hence, the following research questions were put forward:

RQ1: To what extent do gender disparities exist among Chinese college students enrolled in STEM majors with respect to their self-perceived employability?

RQ2: To what degree does gender play a role in the influence of college experiences on individuals’ self-perceived employability?

RQ3: How do the classifications of universities contribute to variations in the impact of college experiences on the self-perceived employability of female Chinese students majoring in STEM fields?

## Methodological research framework

This study utilized survey data from the 2018 Chinese College Student Survey (CCSS), which was adapted from the National Survey of Student Engagement (NSSE) developed in the United States and is the largest survey for students in colleges and universities in China. The survey data, collected between May and September 2018, underwent data cleaning from October to December of the same year. We specifically opted for the 2018 data due to minor adjustments in the questionnaire items in subsequent years, which did not align with our requirements for items related to self-perceived employability.

The analytic dataset utilized included 59,066 undergraduate students in STEM majors in 30 full-time HEIs, including 4 Tier-1 universities, 9 Tier-2 universities and 17 Tier-3 universities. In phase I, universities across China were chosen based on type and geographic area. Subsequently, in phase II, students were randomly selected by grade and major within each university. In each institution, sampling weights were applied according to the students’ grade and major to ensure the representativity of the sample. A summary of the participants’ characteristics is shown in Table 1.

**Table 1:** Participant characteristics.

Variable	Subgroup	Number	Percent (%)
Gender	Male	40,086	67.87
	Female	18,980	32.13
Grade	Freshman	11,858	20.08
	Sophomore	17,060	28.88
	Junior	16,618	28.13
	Senior	13,530	22.91
Ethnicity	Han	53,214	90.09
	Minorities	5,852	9.91
University type	Tier 1 (985 project)	3,567	6.04
	Tier-2 (211 project)	24,483	41.45
	Tier 3 (Others)	31,016	52.51

To test the hypotheses, we examined the effect of female students’ college activities on self-perceived employability (SPE) in all three tiers of universities. All the dependent variables and independent variables are listed in Appendix 1. The dependent variable SPE refers to students’ confidence in their personal abilities and consists of 12 items grouped into one factor. An example item is ‘I have adequate knowledge and skills to fit my future job’. The reliability coefficients of the factor (Cronbach’s α) were higher than 0.8. We conducted exploratory factor analysis, and the sampling adequacy measure (KMO) was 0.92 (>0.9), and Bartlett’s test with *p* < 0.001 indicated the suitability of the self-perceived employability scale for factor analysis. Utilizing Principal Component Analysis, we extracted one factor. The results revealed a cumulative explanatory variation of 64.82%, with all items displaying a maximum factor loading greater than 0.5.

The Independent variable, students’ perceived gain from college experience, consisted of three factors: curriculum experience, extracurricular experience, and faculty supportive activities (see Table 2). Curriculum experience refers to students’ learning experience of the outlined objectives, content, and assessment required to earn a specific academic degree (McCaslin and Good, 1996). In this study, three questions were used to measure students’ perception of gains from the formal curriculum: to what extent does the professional curriculum empower students’ job seeking, how challenging are the professional courses, and to what extent does the formal curriculum enhance students’ ability to solve practical problems? Extracurricular experience refers to a set of momentary experiences that students aggregate and internalize with varying degrees of awareness and satisfaction that are deliberately arranged by the university or students in addition to the formal curriculum (McCaslin and Good, 1996). This study measured students’ perception of gains from extracurricular activities using five categories of activities closely related to career development: frequency of internship, extracurricular English language learning, academic competition, certificates, and research participation. Faculty supportive activities refer to the informational, instrumental, emotional, or appraisal support given by teachers in any environment (Lei et al., 2018). This study used student–faculty interaction frequency to measure students’ perception of faculty supportive activities. Notably, there are two kinds of special faculty members in the Chinese higher education system: college counselors (*fu dao yuan*) and mentors (*ban zhu ren*). Counselors (*fu dao yuan*) who are senior-year students or new graduates serve as role models or ideological mentors who take care of students’ college affairs. Because of their similar age, they frequently develop close relationships with undergraduate students (Wang and Xie, 2013). Mentors are faculty members who provide academic and career guidance for students. The reliability coefficients of the three factors of students’ perception of college experience were all higher than 0.6. Exploratory factor analysis was conducted on the scale of students’ perception of college experience, KMO = 0.76, Bartlett’s test *p* < 0.001, showing that the scale was suitable for factor analysis. Three factors were extracted by PCA and the varimax method. The results showed that the cumulative explanatory variation of the three factors was 63.39%, and the maximum factor loading of all items was greater than 0.5.

**Table 2:** Instruments description.

	Factors	Variables	Items
Dependent Variable	SPE (self-perceived employability)	Self-perceived employability	12
Independent Variables	Curriculum experience	The degree to which the professional curriculum enhances job seeking	1
		Academic challenge	1
		The degree to which the curriculum improves the ability to solve problems	1
	Extracurricular experience	Internship experience	1
		English language learning outside class	1
		Research experience	1
		Participating in academic competitions	1
		Application for professional qualifications/certificates	1
	Faculty supportive activities	Student–faculty interaction: with course instructors	1
		Student–faculty interaction: with mentors	1
		Student–faculty interaction: with *fudaoyuan*	1

The quantitative analysis utilized survey data from 50,699 students in STEM majors and consisted of two parts: an ANOVA that compared the SPE and college experience between female students and male students in STEM majors and a heterogeneity analysis that examined the ways the tier of the university shapes the impact of the college experience on female students.

In phase II of the research, focus group interviews were conducted with female STEM students in the three tiers of universities to understand their views on SPE. We selected participants who were in their senior year of undergraduate study or the first year of graduate school to ensure that they had thoughts or experience related to career plans after college graduation. Six focus groups were formed: 2 groups consist students from a Tier-1 university, 2 groups consist students from two Tier-2 universities, and 2 groups consists students from two Tier-3 universities (Table 3). The authors acted as moderators. After guided the participants with structured questions, the authors encouraged the participants to express themselves freely on topics of interest.

**Table 3:** Data about participants of focus group interviews.

Group	Participants
Tier-1G01	3 first-year master’s students from T University majoring in automation
Tier-1G02	4 senior students from T University majoring in chemistry and chemical engineering
Tier-2G01	4 senior students from W University majoring in electronic engineering and material sciences
Tier-2G02	3 first-year master’s students from L University majoring in mechanical engineering and biology
Tier-3G01	3 senior students from Q University majoring in civil engineering, photonics engineering, and engineering mechanics
Tier-3G02	3 senior students from H University majoring in software engineering, automotive engineering, and material sciences

## Findings

The findings of this study shed light on the self-perceived employability (SPE) of male and female students in different university tiers, revealing nuanced gender disparities and their impact on career outcomes. Table 4 presents a comprehensive overview, illustrating that SPE varied not only by gender but also by university tier. Further analysis through ANOVA unveiled intricate interaction effects between gender and university tier on SPE. In exploring specific aspects of university experiences, gender differences were found to be statistically insignificant in curriculum and some extracurricular domains across all university tiers. The results of a focus group interview, providing valuable insights into the perspectives of female STEM students across different university tiers and their encounters with gender-based challenges in the pursuit of employment opportunities.

**Table 4:** Mean values of variables in different subgroups.

Factors	Variables	T1-M	T1-F	T2-M	T2-F	T3-M	T3-F	*p* value
Self-perceived employability	SPE	60.931	55.760	61.393	57.463	58.973	53.904	*p* < 0.05
Curriculum experience	Professional curriculum-job seeking	61.466	60.767	60.376	60.592	59.541	60.213	*p* > 0.05
	Academic challenge	61.984	63.521	60.088	62.927	59.618	61.427	*p* > 0.05
	Curriculum – solving problems	77.924	80.185	75.104	77.047	74.863	76.600	*p* > 0.05
Extracurricular experience	Internship	24.710	20.030	28.410	25.550	33.020	29.980	*p* < 0.05
	English language learning outside class	16.950	20.850	14.940	18.690	14.150	14.770	*p* < 0.05
	Academic competitions	29.020	29.180	23.060	27.440	22.280	22.630	*p* < 0.05
	Certificates	29.660	30.700	31.780	41.910	31.630	39.390	*p* < 0.05
	Research experience	8.670	12.460	7.790	9.430	6.610	5.840	*p* < 0.05
Faculty supportive activities	Student–faculty interaction: instructors of courses	34.183	30.491	38.341	33.291	43.335	38.796	*p* < 0.05
	Student–faculty interaction: class teacher	33.848	29.624	37.166	32.056	40.785	34.748	*p* < 0.05
	Student–faculty interaction: fudaoyuan	30.684	24.564	34.508	28.163	38.487	31.416	*p* < 0.05

### Self-perceived employability of male and female students

Mean values presented in Table 4, provide a measure of central tendency, offering insight into the average level of SPE across different groups. Table 4 shows that SPE varied by gender and by university tier. Students in Tier-2 universities scored higher in SPE compared to those from the other two groups. Moreover, female students’ SPE scores were significantly lower (*p* < 0.001) than those of male students in the same university type. The ANOVA results, as shown in Table 4, reveal that the interaction effects of gender and university tier on SPE were complex. Considering the large sample size, we employed the Bonferroni correction method to control for Type I error inflation due to multiple comparisons. All *p*-values reported in Table 4 were adjusted by multiplying them by the number of comparisons conducted.

In examining university settings across all tiers, gender differences were found to be non-significant in the formal curriculum. However, within extracurricular activities, notable distinctions emerged. Female students exhibited greater enthusiasm for self-directed English study outside of class and actively participated in research, whereas their male counterparts reported more internship experiences. Interestingly, females attending Tier-2 and Tier-3 universities displayed a heightened interest in acquiring certificates compared to their male peers, possibly reflecting a proactive effort among female students to validate and demonstrate their competencies in the competitive labor market.

Moreover, females from Tier-2 and Tier-3 universities reported a higher frequency of student–faculty interactions than those in Tier-1 universities. Despite this, female students, across all tiers, reported significantly lower interaction frequencies with faculty compared to their male counterparts. This gender gap may be partly attributed to concealed gender discrimination, as highlighted in previous studies (Hall and Sandler, 1982; Sax, 2008; Liu et al., 2011; Cohen, 2018). Additionally, the underrepresentation of female faculty in universities contributes to the observed lower frequency of interactions between female students and faculty members.

### Effects of college experience in different universities on self-perceived employability

As shown in Table 5, curriculum, extracurricular activities and faculty supportive activities have significant positive impact on SPE. The effect of the curriculum on SPE showed no significant differences between female STEM students among various universities. Nevertheless, it is worth noting that the curriculum remains the most influential factor on SPE among all of the independent variables. The STEM professional curriculum, in particular, is a crucial pathway to empower female STEM students. Previous studies have found that perceptions of belonging to this major and to the classroom environment support female students to form an engineering identity, and a lack of belongingness may be most prevalent among students who switch out of STEM fields (Foor et al., 2007; Verdín, 2021).

**Table 5:** Impact of college experience on SPE.

		*b*(se)	*t*
Curriculum experience	Professional curriculum-job seeking	0.213 (0.005)	41.71***
	Academic challenge	0.055 (0.005)	11.09***
	Curriculum – solving problems	0.097 (0.005)	19.65***
Extracurricular experience	Internship	0.013 (0.002)	5.07***
	English language learning outside class	0.034 (0.003)	11.11***
	Academic competitions	0.023 (0.003)	8.43***
	Certificates	0.005 (0.002)	2.26*
	Research experience	0.025 (0.004)	6.04***
Faculty supportive activities	Student–faculty interaction: instructors of courses	0.050 (0.005)	9.56***
	Student–faculty interaction: class teacher	0.040 (0.007)	5.81***
	Student–faculty interaction: fudaoyuan	0.087 (0.007)	13.31***
Controlled variables		Yes	
Sample size		18,953	
*R*-squared adjusted		0.3251	
*F* value		829.46***	

*p < 0.05, **p < 0.01, ***p < 0.001.

We measured several extracurricular activities closely related to employability development, including the frequency of participation in academic competitions, the duration of internships and part-time jobs, and the frequency of professional qualification certificates. Different extracurricular activities had varied impacts on female STEM students in different types of universities. In terms of English self-study and professional qualification certificates, the positive effect on female students in Tier-2 and Tier-3 universities was significantly larger than that of Tier-1 universities. Moreover, the positive effect of research participation of Tier-3 female students was significantly larger than that of Tier-1 and Tier-2 universities. One possible explanation for these findings is that Tier-2 and Tier-3 female students may believe that their university reputation is of limited help in employment, so they may need to enroll in better universities for graduate studies or study abroad. Therefore, English language learning and research participation may be highly beneficial to achieving these goals.

Social support refers to the assistance that an individual can access through social ties with other individuals, groups, and the broader community. In this study, we measured social support through student–faculty interactions. Interestingly, the positive effect of student–faculty interaction on the SPE scores of female students in Tier-1 and Tier-3 universities was significantly lower than that of students in Tier-2 universities. This difference in student–faculty interaction may be related to the positioning of Tier-2 universities in China’s higher education system. The resources and reputation of Tier-2 universities are far better than those of Tier-3 universities, while the employment orientation is stronger than that of Tier-1 universities. The goal of Tier-1 universities is to ‘lay a solid foundation for China to become a moderately developed country’ and to cultivate research-oriented talent who can serve the country’s development (MOE, 2004). The goal of Tier-2 universities is to ‘solve the major problems of economic construction and social development.’ Compared with the political and social significance of Tier-1 universities, Tier-2 universities emphasize economic factors to a greater extent (Xu and Wang, 2007). Conversely, Tier-2 universities, with a focus on addressing major economic and social challenges, align more closely with employment-oriented objectives. The superior resources and reputation of Tier-2 institutions compared to Tier-3 counterparts likely contribute to a more robust infrastructure for fostering meaningful student–faculty interactions. This heightened emphasis on employment outcomes in Tier-2 universities could explain the observed stronger positive effect of these interactions on the SPE scores of female students (Table 6).

**Table 6:** Moderated effect size of female students in different subgroups.

		Female in Tier-1	Female in Tier-2	Female in Tier-3
Curriculum experience	Professional curriculum-job seeking	0.000 (reference)	0.011	0.003
	Academic challenge	0.000 (reference)	−0.022	−0.031
	Curriculum- solving problems	0.000 (reference)	0.031	0.016
Extracurricular experience	Internship	0.000 (reference)	0.007	0.001
	English language learning outside class	0.000 (reference)	0.022**	0.037***
	Academic competitions	0.000 (reference)	0.002	0.013
	Certificates	0.000 (reference)	0.011*	0.013*
	Research experience	0.000 (reference)	0.021	0.068***
Faculty supportive activities	Student–faculty interaction: instructors of courses	0.000 (reference)	0.014*	0.013
	Student–faculty interaction: class teacher	0.000 (reference)	0.004	0.007
	Student–faculty interaction: fudaoyuan	0.000 (reference)	0.014*	0.011

*p < 0.05, **p < 0.01, ***p < 0.001.

### Results of the focus group interview

#### Female STEM students at Tier-1 universities: the individual gap among female students is wider than the female–male gap

Female participants from the sampled Tier-1 university stated that the university reputation was a stepping stone for them to seek jobs, but they did not think their university experience offered much help in raising their individual SPE. Female STEM students at such universities had relatively high SPE and believed that gender differences were much smaller than individual differences in general. For example, a master’s student at a Tier-1 university (Tier-1G01) said she felt no different from her male peers. She elaborated as follows:

Because of the good reputation of my school, an employer will quickly give me a chance. On the other, people may have higher expectations of me. I worry that my work ability is not worthy of the school brand, so I often feel less confident. But in general, SPE comes from individual ability, and I do not think I’m any different from my male peers. (Tier-1 G01)

Interestingly, female students at a Tier-1 university from other majors in the senior year (Tier-1G02) showed considerable confidence in their personal capabilities. A student in chemical engineering said,

My admission to this university proves that I can do my job to some extent. Based on my personal experience, my colleagues tend to ignore gender and will not take care of me just because I am a girl. (Tier-1G02)

As a whole, female students were not highly aware of the gender gap during the job-seeking process, and they were enthusiastic about improving themselves to enhance their SPE. Not all Tier-1 female STEM students were so confident, however. Depending on their personal experiences, their views on gender differences were somewhat subtle. Female students who had already been exposed to the job-seeking process, such as summer internships, were more pessimistic and perceived the plight of STEM women indirectly by observing the career development of their female colleagues, while female students who were not exposed to the workplace had difficulty perceiving the existence of a gender gap.

#### Female STEM students at Tier-2 universities: the gender gap demands extra effort from women

For female students at Tier-2 universities, the issue of sexism becomes more subtle and complex. Tier-2 universities have a brand effect in the job market, and quality teaching helps students acquire competitive knowledge and skills. However, compared to their male peers, female STEM students feel being discriminated to a certain degree. They actively participate in various activities to enhance their resumes to prove that they can do better than male students and are not less competitive than male students. Some female students from Tier-2 universities (Tier-2G01, Tier-2G02) reported that employers made no secret of their preference for men in the recruitment process. A student said,

In some job-hunting processes, companies will not hesitate to recruit male students rather than female students with the same qualifications. I often feel helpless because even if the girl is better than the boy in many ways, the company will hire the boy. (Tier-2G01)

Another female student echoed this point and said,

If I hadn’t attended last fall’s recruiting season, I would have thought there was no gender gap. Overall, the jobs we found were good but generally worse than those of men of the same major and level. (Tier-2G02)

Compared to peers at Tier-1 universities, female students from Tier-2 universities were fully aware of the gender gap and were resentful that they did not have the same opportunities as their male peers.

#### Female students at Tier-3 universities: individual efforts are negligible when facing sexism

Female students in Tier-3 universities recognized the apparent gender discrimination in workplaces. They felt female students’ efforts were meaningless and that the gender differences in employment were far greater than individual differences. As a result, many female STEM students either chose to pursue postgraduate education in a higher-ranked university or chose a career in non-STEM fields. An engineering student studying at a Tier-3 university said,

In many tests, such as those requiring rote memorisation or a lot of practice, girls are better than boys. But in the actual interview process, employers will not look at these scores and certificates and directly reject girls because of gender. (Tier-3G01)

For female students at Tier-3 universities, the gender gap is no longer just a negative factor that produces extra difficulties in job hunting; it changes their career choices. A student from a Tier-3 university elaborated,

Male preference has become the basic rule in our job-hunting process, and our efforts to improve grade points or to obtain certificates on campus seem to be meaningless for job hunting. Most of the time, our efforts are just to change majors or to further our education. (Tier-3G02)

## Discussion

The findings of this study reveal the differences in self-perceived employability of female students in STEM majors in three tiers of universities in China.

One of the major findings has confirmed that the hierarchical higher education system has positioned female STEM students in the bottom universities in a very difficult position. There is a steep hierarchy in the Chinese higher education system that leads to varied college experience and different SPE of female students from universities of different tiers. In contrast to the higher education system in Western countries, Chinese universities are deeply embedded in their political system and rely on the government for resources (Zha et al., 2017). Marginson (2011) developed the concept of the ‘Confucian model’ to define the close link between academics and national governance in China and other Asian countries. In Confucian society, the reputation of a university is determined by the government-assigned ‘tier’ regardless of the real outcomes, and employers believe that enrolling in a Tier-1 university counts more than graduating with good GPA from a Tier-3 university (Han and Guo, 2015). According to our quantitative analysis, formal curriculum experience is the leading factor in STEM students’ SPE, but academic performance on campus may be covered up by the tier of the university. Some scholars have criticized that the main function of China’s higher education system has been reduced to simple social stratification (Wu and Guo, 2018; Luo et al., 2021), which significantly affects college students’ expectations for their future career paths and their college experience.

We found that female students in STEM majors in Tier-2 and Tier-3 universities made tremendous efforts to develop their resumes through a wide array of extracurricular activities, reflecting their awareness of their dual disadvantaged position in the job market with regard to gender and institutional reputation. Female students from Tier-3 universities have a much stronger motivation to prove their capability through various kinds of certificates or qualifications, in part to compensate for the recognition that a diploma from their own universities cannot provide much help in getting them good jobs. However, unlike female graduates from Tier-1 or Tier-2 universities who are exposed to a wider range of science and engineering jobs, female students in Tier-3 universities are more likely to be matched to more labor-intensive jobs (such as processing and assembly), which puts them in more disadvantaged positions when competing with male applicants. In addition, women’s careers are easily interrupted by childbirth, resulting in the phenomenon of ‘leaving early’ in their careers. In China, female students in Tier-1 universities have more opportunities to join government organizations, state-owned enterprises, and public institutions, whereas most female STEM students in Tier-3 universities are employed outside the state-owned system. They are more likely to be forced to resign after giving birth and find it difficult to return to work after lactation (Li, 2016; Ding and Xie, 2020).

Another major finding of this study highlights the fact that gender has not gained sufficient attention in Chinese universities regardless their tiers in the stratified higher education system. This study finds that faculty members and college students have insufficient gender awareness, and universities do not have adequate agency to narrow the gender gap. Some gender researchers in China have analyzed the reasons for the lack of gender awareness of contemporary Chinese people from a historical perspective. They believe that in contrast to the feminist movement in the West where women pursued their own rights, China’s ‘women’s liberation’ is a byproduct of China’s social revolution, and gender equality in law and policy is ahead of the public’s personal consciousness (Yu, 2011). We found that female students had a significant disadvantage compared with their male peers in terms of informal curriculum experience and the frequency of student–faculty interaction, but universities rarely offer specific measures to narrow the gender gap. In addition, given the gender discrimination in employment commonly faced by female STEM students, universities rarely provide sufficient support through employment guidance in advance, so female students only realise the existence of gender differences when they participate in the job-seeking process.

The results of this study have several important implications for theory, research, policy, and practice. First, there is a need to reflect on and expand the use of human capital theory of gender disparity in the studies of higher education in the different national contexts. Human capital theory posits that engaging in career-related activities can enhance the self-perceived employability of female STEM students. As some scholars have found, female STEM students in lower-tier universities need academic competitions and professional qualification certificates as human capital to prove their work ability due to the weak signaling role of their universities in the labor market (Zhang and Zhen, 2011; Zhao, 2011). However, the outcomes of this research reveal a departure from this theory, like a few studies conducted in the context of other developing countries (Dao, 2012; Fatima, 2013). In the context of China and similar developing economies, the established dynamic mechanism linking career-related activities to improved self-perceived employability appears to be attenuated. The physical power gap becomes a prominent factor shaping female students’ self-perceived employability in developing nations, which overshadows the impact of college experiences, potentially leading to a scenario where female students find themselves disheartened about investing in career related activities.

With regard to research, the academic significance of this research lies in its innovative approach, providing an intersectional perspective that delves into the underrepresentation of female students in STEM disciplines through the lens of both gender and university tier. This unique analytical framework offers a complement to previous institutional theories by uncovering the intricate dynamics shaping the experiences of female STEM students within the hierarchical structure of the Chinese higher education system. While prior research has primarily focused on the institutional factors influencing educational and career outcomes, our study enhances this perspective by elucidating how the stratification within the Chinese higher education system intertwines with gender dynamics, thereby influencing the self-perceived employability of female STEM students (Dheer et al., 2019; Gaweł and KRSTIĆ, 2021). This adds to scholarship on gender inequality in China, which previously focused on the societal effects on female students’ educational outcomes and career choices rather than on female students’ reflexive experience. By analysing female students’ experience and self-perceived employability in different tiers of universities, this study captured a nuanced understanding of female students in STEM majors in Chinese higher education, which extends the generalizability of the results of research on females in STEM fields conducted in Western countries and documents the current landscape and challenges for females in STEM majors in China for future international comparative research. Additionally, the impact of national culture, particularly gender culture, on female students’ experience and employability can be revisited in future studies to identify the reasons for the differences found in female students’ self-perceived employability and college experience across countries.

In terms of implications for policy, the results highlight the varied impact of college experience on female students in STEM majors in universities of different tiers. The world-class university movement of China stratifies Chinese universities, which has a large influence on the ways the Chinese job market perceives and hires university graduates and strongly shapes college students’ perceived employability. The results of this study can help policymakers critically reflect on the potential ramifications of the university stratification of the Chinese higher education system and reduce the negative effects of the stratification for certain groups of students accordingly in broader society.

In terms of implications for practice, this research provides valuable evidence for academic and student affairs practitioners as well as university administrators and policymakers to understand how university activities affect female students who enroll in traditionally male-dominated fields of study. By highlighting the importance of enhancing female students’ self-perceived employability, this research hopes to encourage universities of different tiers to provide a wider array of work-integrated learning opportunities for female students and invite faculty members, mentors, and staff to spend more time interacting with female students in STEM majors.

## Conclusion

The findings of this study unfold distinctive patterns across Tier-1, Tier-2, and Tier-3 universities, shedding light on the varied experiences and challenges faced by female STEM students within this stratified higher education system. The findings highlight the importance of recognizing that, within a hierarchical higher education system, female students may perceive different impacts based on the position of their university. It is important to have context-specific considerations in shaping strategies for empowering and advancing the careers of female STEM students in developing countries or countries with stratified higher education system.

While this study provides valuable insights into the self-perceived employability of female students in STEM majors across different tiers of universities in China, it is important to acknowledge certain limitations that may impact the generalizability and interpretation of the findings. First, this study provides a snapshot of the situation at a specific point in time. Given the dynamic nature of societal and educational systems, the findings may not capture potential changes or developments that could occur in the future. Second, this study primarily focuses on the intersection of gender and university tier, while did not bring other potential intersectional factors into the research such as socioeconomic status or regional differences. Future research will explore these intersectional factors so as to provide a more comprehensive understanding of the challenges faced by female STEM students. Third, faculty perspectives are not extensively explored in this study. Including faculty viewpoints could provide a richer exploration of the factors influencing self-perceived employability.

## Data availability statement

The datasets presented in this article are not readily available because the datasets generated and/or analyzed during the current study are not publicly available to protect the participants’ privacy. Requests to access the datasets should be directed to LZ, zhoul22@mails.tsinghua.edu.cn.

## Ethics statement

The studies involving humans were approved by Institute of Education, Tsinghua University. The studies were conducted in accordance with the local legislation and institutional requirements. The participants provided their written informed consent to participate in this study. Written informed consent was obtained from the individual(s) for the publication of any potentially identifiable images or data included in this article.

## Author contributions

WW: Conceptualization, Project administration, Supervision, Writing – review & editing, Writing – original draft. LZ: Formal analysis, Methodology, Writing – original draft, Writing – review & editing. DH: Project administration, Writing – original draft, Writing – review & editing. MZ: Data curation, Methodology, Writing – review & editing. ZY: Data curation, Methodology, Writing – review & editing. XT: Writing – review & editing.
