# Supplementary material for: The impact of college experience on female students’ self-perceived employability in STEM majors
Source: Front Psychol. 2023 Dec 21;14:1282934. doi: 10.3389/fpsyg.2023.1282934 (PMC10768181; doi:10.3389/fpsyg.2023.1282934)
Supplement: Supplementary file 1 [file Table_1.docx]

Appendix 1 Items used in the questionnaire

A1 Self-Perceived Employability

A1.1 To what extent do you agree? When looking for a job after graduation, the reputation of our university serves as intangible capital.

(1) totally disagree, (2) disagree, (3) agree, (4) totally agree

A1.2 To what extent do you agree? Graduates from our major are in high demand in the job market.

(1) totally disagree, (2) disagree, (3) agree, (4) totally agree

A1.3 To what extent do you agree? I possess the key knowledge and skills required for employment in the relevant field.

(1) totally disagree, (2) disagree, (3) agree, (4) totally agree

A1.4 To what extent do you agree? I place great importance on my professional studies.

(1) totally disagree, (2) disagree, (3) agree, (4) totally agree

A1.5 To what extent do you agree? Our university excels in the field of my major.

(1) totally disagree, (2) disagree, (3) agree, (4) totally agree

A1.6 To what extent do you agree? I have a comprehensive and clear understanding of the types, quantity, and geographical distribution of potential employers in my future professional field.

(1) totally disagree, (2) disagree, (3) agree, (4) totally agree

A1.7 To what extent do you agree? In the future employment sector, I have consciously established relationships with relevant institutions and individuals for future job requirements.

(1) totally disagree, (2) disagree, (3) agree, (4) totally agree

A1.8 To what extent do you agree? I am confident that I will stand out in future employment interviews and selection processes.

(1) totally disagree, (2) disagree, (3) agree, (4) totally agree

A1.9 To what extent do you agree? Finding a job in the employment sector corresponding to my major is not difficult.

(1) totally disagree, (2) disagree, (3) agree, (4) totally agree

A1.10 To what extent do you agree? Based on my abilities, qualities, and experiences, achieving success in the workplace is only a matter of time.

(1) totally disagree, (2) disagree, (3) agree, (4) totally agree

A1.11 To what extent do you agree? Generally speaking, the current job market is favorable.

(1) totally disagree, (2) disagree, (3) agree, (4) totally agree

A1.12 To what extent do you agree? I have the ability to solve practical problems in this field.

(1) totally disagree, (2) disagree, (3) agree, (4) totally agree

A2 Curriculum experience

A2.1 To what extent do you agree? Your curriculum offers you professional knowledge and skills in this field.

(1) totally disagree, (2) disagree, (3) agree, (4) totally agree

A2.2 To what extent do you agree? Your curriculum is challenging and inspires you to keep working hard.

(1) totally disagree, (2) disagree, (3) agree, (4) totally agree

A2.2 To what extent do you agree? Your curriculum improves your ability to solve practical problems.

(1) totally disagree, (2) disagree, (3) agree, (4) totally agree

A3 Extracurricular experience

A3.1 Are you involved or planning to be involved in: internship

(1) never and will not, (2) have not decided, (3) plan to do, (4) have done and will continue

A3.2 Are you involved or planning to be involved in: English learning outside class

(1) never and will not, (2) have not decided, (3) plan to do, (4) have done and will continue

A3.3 Are you involved or planning to be involved in: academic competitions

(1) never and will not, (2) have not decided, (3) plan to do, (4) have done and will continue

A3.4 Are you involved or planning to be involved in: certificates

(1) never and will not, (2) have not decided, (3) plan to do, (4) have done and will continue

A3.5 Are you involved or planning to be involved in: research experience

(1) never and will not, (2) have not decided, (3) plan to do, (4) have done and will continue

A4 Faculty supportive activities

A4.1 How often do you discuss your career plans with your teachers? With course instructors

(1) never, (2) sometimes, (3) often (4) very often

A4.2 How often do you discuss your career plans with your teachers? With class teachers

(1) never, (2) sometimes, (3) often (4) very often

A4.3 How often do you discuss your career plans with your teachers? With fudaoyuan

(1) never, (2) sometimes, (3) often (4) very often
